# Ethnic differences in SARS-CoV-2 vaccine hesitancy in United Kingdom healthcare workers: Results from the UK-REACH prospective nationwide cohort study

**DOI:** 10.1016/j.lanepe.2021.100180

**Published:** 2021-07-19

**Authors:** Katherine Woolf, I Chris McManus, Christopher A Martin, Laura B Nellums, Anna L Guyatt, Carl Melbourne, Luke Bryant, Mayuri Gogoi, Fatimah Wobi, Amani Al-Oraibi, Osama Hassan, Amit Gupta, Catherine John, Martin D Tobin, Sue Carr, Sandra Simpson, Bindu Gregary, Avinash Aujayeb, Stephen Zingwe, Rubina Reza, Laura J Gray, Kamlesh Khunti, Manish Pareek, UK-REACH Study Collaborative Group

**Affiliations:** a University College London Medical School, United Kingdom; b Department of Respiratory Sciences, University of Leicester, United Kingdom; c Department of Infection and HIV Medicine, University Hospitals of Leicester NHS Trust, United Kingdom; d Division of Epidemiology and Public Health, School of Medicine, University of Nottingham, United Kingdom; e Department of Health Sciences, University of Leicester, United Kingdom; f Oxford University Hospitals NHS Foundation Trust, United Kingdom; g University Hospitals Leicester NHS Trust, Leicester Royal Infirmary, United Kingdom; h General Medical Council, United Kingdom; i Nottinghamshire Healthcare NHS Foundation Trust, United Kingdom; j Lancashire Clinical Research Facility, Royal Preston Hospital, United Kingdom; k Respiratory department, Northumbria Specialist Emergency Care Hospital, United Kingdom; l Research and Development Department, Berkshire Healthcare NHS Foundation Trust, United Kingdom; m Derbyshire Healthcare NHS Foundation Trust Centre for Research and Development, Kingsway Hospital site, United Kingdom; n Diabetes Research Centre, University of Leicester, United Kingdom

## Abstract

**Background:**

In most countries, healthcare workers (HCWs) represent a priority group for vaccination against severe acute respiratory syndrome coronavirus-2 (SARS-CoV-2) due to their elevated risk of COVID-19 and potential contribution to nosocomial SARS-CoV-2 transmission. Concerns have been raised that HCWs from ethnic minority groups are more likely to be vaccine hesitant (defined by the World Health Organisation as refusing or delaying a vaccination) than those of White ethnicity, but there are limited data on SARS-CoV-2 vaccine hesitancy and its predictors in UK HCWs.

**Methods:**

Nationwide prospective cohort study and qualitative study in a multi-ethnic cohort of clinical and non-clinical UK HCWs. We analysed ethnic differences in SARS-CoV-2 vaccine hesitancy adjusting for demographics, vaccine trust, and perceived risk of COVID-19. We explored reasons for hesitancy in qualitative data using a framework analysis.

**Findings:**

11,584 HCWs were included in the cohort analysis. 23% (2704) reported vaccine hesitancy. Compared to White British HCWs (21.3% hesitant), HCWs from Black Caribbean (54.2%), Mixed White and Black Caribbean (38.1%), Black African (34.4%), Chinese (33.1%), Pakistani (30.4%), and White Other (28.7%) ethnic groups were significantly more likely to be hesitant. In adjusted analysis, Black Caribbean (aOR 3.37, 95% CI 2.11 - 5.37), Black African (aOR 2.05, 95% CI 1.49 - 2.82), White Other ethnic groups (aOR 1.48, 95% CI 1.19 - 1.84) were significantly more likely to be hesitant. Other independent predictors of hesitancy were younger age, female sex, higher score on a COVID-19 conspiracy beliefs scale, lower trust in employer, lack of influenza vaccine uptake in the previous season, previous COVID-19, and pregnancy. Qualitative data from 99 participants identified the following contributors to hesitancy: lack of trust in government and employers, safety concerns due to the speed of vaccine development, lack of ethnic diversity in vaccine studies, and confusing and conflicting information. Participants felt uptake in ethnic minority communities might be improved through inclusive communication, involving HCWs in the vaccine rollout, and promoting vaccination through trusted networks.

**Interpretation:**

Despite increased risk of COVID-19, HCWs from some ethnic minority groups are more likely to be vaccine hesitant than their White British colleagues. Strategies to build trust and dispel myths surrounding the COVID-19 vaccine in these communities are urgently required. Emphasis should be placed on the safety and benefit of SARS-CoV-2 vaccination in pregnancy and in those with previous COVID-19. Public health communications should be inclusive, non-stigmatising and utilise trusted networks.

**Funding:**

UKRI-MRC and NIHR.

**Research in context**

**Evidence before this study**

We searched Pubmed using the following search terms ((COVID-19).ti,ab OR (SARS-CoV-2).ti,ab) AND ((vaccine).ti,ab OR (vaccination).ti,ab OR (immunisation).ti,ab)) AND ((healthcare worker).ti,ab OR (health worker).ti,ab OR (doctor).ti,ab OR (nurse).ti,ab OR (healthcare professional).ti,ab)) AND ((hesitancy).ti,ab OR (refusal).ti,ab OR (uptake).ti,ab)). The search returned 60 results, of which 38 were excluded after title and abstract screening, 11 studies were not conducted in a population of healthcare workers, 20 did not present data on vaccine intention or uptake, 5 were related to vaccines other than the SARS-CoV-2 vaccine, 1 was unrelated to vaccination and 1 had been withdrawn. The 22 remaining articles were survey studies focussed on SARS-CoV-2 vaccine intention in healthcare workers. Estimates of SARS-CoV-2 vaccine acceptance varied widely from 27•7% - 94•5% depending on the country in which the study was performed, and the occupational group studied. Only 2 studies (both conducted in the USA) had a sample size greater than 10,000. Most studies found females, non-medical healthcare staff and those refusing influenza vaccine to be more likely to be hesitant. There was conflicting evidence about the effects of age and previous COVID-19 on hesitancy. Only 3 studies (all from the USA), presented data disaggregated by ethnicity, all finding Black ethnic HCWs were most likely to be hesitant. Common themes amongst studies that investigated reasons for vaccine hesitancy were concerns about safety of vaccines, fear of side effects and short development timeframes. We did not find any studies on SARS-CoV-2 vaccine hesitancy in UK healthcare workers in the published literature.

**Added value of this study**

This study is amongst the largest SARS-CoV-2 vaccine hesitancy studies in the literature. It is the largest study outside the USA and is the only study in UK HCWs. Our work focusses on the association of ethnicity with vaccine hesitancy, and we are the first study outside the USA to present results by ethnic group. The large number of ethnic minority HCWs in our study allows for examination of the outcome by more granular ethnicity categories than have previously been studied, allowing us to detect important differences in vaccine hesitancy levels within the broad White and Asian ethnic groupings. Our large sample size and the richness of our cohort study dataset allows us to control for many potential confounders in our multivariable analysis, and provide novel data on important potential drivers of hesitancy including discrimination, COVID-19 conspiracy beliefs, religion/religiosity and personality traits. Additionally, we combine quantitative with qualitative data providing a deeper understanding of the drivers of hesitancy and potential strategies to improve vaccine uptake in HCWs from ethnic minority communities.

**Implications of all the available evidence**

Around a quarter of UK healthcare workers reported SARS-CoV-2 vaccine hesitancy. In accordance with previous studies in other countries, we determined that female sex and lack of influenza vaccine in the previous season were important predictors of SARS-CoV-2 vaccine hesitancy in UK HCWs, although in contrast to most studies in the published literature, after adjustment we do not demonstrate differences in hesitancy levels by occupational role. Importantly, previous literature provides conflicting evidence of the effects of age and previous SARS-CoV-2 infection on vaccine hesitancy. In our study, younger HCWs and those with evidence of previous COVID-19 were more likely to be hesitant. This study provides novel data on increased hesitancy levels within Black Caribbean, Mixed White and Black Caribbean, Black African, Chinese, Pakistani and White Other ethnic groups. Mistrust (of vaccines in general, in SARS-CoV-2 vaccines specifically, in healthcare systems and research) and misinformation appear to be important drivers of hesitancy within HCWS in the UK. Our data indicate that despite facing an increased risk of COVID-19 compared to their White colleagues, UK HCWs from some ethnic minority groups continue to exhibit greater levels of SARS-CoV-2 vaccine hesitancy. This study provides policy makers with evidence to inform strategies to improve uptake.

Alt-text: Unlabelled box

## 1 Introduction

An unprecedented global research effort has resulted in effective vaccines against the causative agent of COVID-19, severe acute respiratory syndrome coronavirus 2 (SARS-CoV-2) [1,2]. Emerging evidence suggests that mass vaccination programmes, which are underway globally, can significantly reduce the incidence of COVID-19 infections, hospitalisations and deaths [3]. The UK Joint Committee on Vaccination and Immunisation (JCVI) have prioritised certain high-risk groups in the UK's vaccination programme, including frontline health and social care staff. There are however concerns about SARS-CoV-2 vaccine hesitancy among healthcare workers (HCWs) [4], [5], [6], [7], [8], [9], [10], [11], [12], [13], and particularly among some ethnic minority groups [14], [15], [16], [17], [18], [19], [20], [21] including ethnic minority HCWs [22,23] despite those groups being disproportionately affected by the pandemic [24,25].

Vaccine hesitancy is defined by the World Health Organisation (WHO) as refusal or delay in vaccine acceptance [26]. Levels of hesitancy towards specific vaccines and/or vaccines in general differ across individuals. Vaccine hesitancy amongst HCWs is especially concerning because it increases risk to the health of the individual HCW, is likely to increase the risk of nosocomial SARS-CoV-2 transmission [27], and may influence patient vaccine uptake [15,28]. Reasons for vaccine hesitancy vary between individuals and by context, geographic location and vaccine, but the WHO's “Three C's model” has identified three areas influencing hesitancy: Convenience (vaccine access), Confidence (trust - in vaccines generally, in their efficacy, in those providing the vaccine, and in those creating vaccine policy), and Complacency (perceived risk of vaccine-related disease) [26,[28], [29], [30]].

The UK's Scientific Advisory Group for Emergencies (SAGE) ethnicity subgroup has identified the following key factors underlying vaccine hesitancy in ethnic minority groups: physical barriers to access; lower trust and confidence in vaccine efficacy and safety, and general lack of trust in healthcare and health research due to structural and institutional racism and discrimination; lower perceived risk; and contextual factors such as gender, education, socioeconomic status and family decision-making [15]. A recent study of predictors of COVID-19 vaccine hesitancy from the UK Household Longitudinal Study found that among vaccine hesitant groups, Black participants were more likely to cite lack of trust in vaccines and worries about unknown future effects of vaccination, whereas Pakistani and Bangladeshi groups were most concerned about side effects as well as unknown future effects [16].

Studies of COVID-19 vaccination intentions and uptake in HCWs since December 2020 show variability in uptake between countries and, as with general populations, variability by occupational and demographic groups [8,22,31,32]. A study in a large UK hospital trust showed that 71% of White staff had been vaccinated against COVID-19 as compared to 59% of South Asian staff and 37% of Black staff. Factors associated with vaccine hesitancy (other than belonging to an ethnic minority group) were younger age, female sex and living in more deprived areas [12].

To date there have been very few large-scale studies of COVID-19 vaccine hesitancy among ethnically diverse HCWs. We undertook an analysis to understand levels of vaccine hesitancy and the factors predicting this in UK HCWs using interim data from the United Kingdom Research study into Ethnicity And COVID-19 outcomes in Healthcare workers (UK-REACH), integrating survey data from a nationwide prospective longitudinal cohort study and qualitative data from HCWs nationwide.

## 2 Methods

### 2.1 Overview

UK-REACH encompasses six studies to understand the impact of COVID-19 on HCWs from diverse ethnic backgrounds. Here we present data from the baseline questionnaire of the UK-REACH prospective cohort study (administered online from 4^th^ December 2020 with interim data downloaded 19^th^ February 2021), and qualitative data from UK-REACH interviews and focus groups (undertaken from December 2020 to March 2021). Both studies took place in healthcare settings in all four nations of the UK with clinical and non-clinical HCWs from diverse ethnic backgrounds; see study protocols for methodological details [33,34].

### 2.2 Prospective nationwide cohort study

Questionnaire design, sampling and baseline questionnaire measures are detailed in the study protocol [33] and data dictionary (https://www.uk-reach.org/data-dictionary).

#### 2.2.1 Study population

All HCWs or ancillary workers in a UK healthcare setting aged 16 or over and/or those registered with one of seven main healthcare regulatory bodies, who responded to an email invitation or who were directly recruited through participating healthcare trusts or open links advertised on social media or in newsletters.

In order to take part, a multi-step process had to be completed. Professional regulators sent out an email and newsletters with a link to the study website to HCWs within their organisations. It is important to note that a survey questionnaire was not sent to every individual. Recipients then had to read the email, navigate to the study website and register to create a profile. Following creation of a study profile, potential participants were asked to read a participant information sheet and then, if they were willing, consent to participate in the study. Only after all these steps were completed was the survey process initiated. This multi-step process, which is common for online/internet web-surveys, allows transparent reporting of participation rates at different points in the multi-step process, rather than as a single response rate, as recommended by the Checklist for Reporting Results of Internet E-Surveys (CHERRIES) [35,36].

#### 2.2.2 Primary outcome measure

We derived the primary outcome, SARS-CoV-2 vaccine hesitancy (binary measure: hesitant versus accepting) from responses to two versions of vaccine questions (VQ1 and VQ2: see Supplementary Fig. 1 for details). Vaccine questions were updated during the recruitment/completion period to reflect rapid inception/evolution of the vaccination programme.

#### 2.2.3 Predictor variables

We selected variables for inclusion based on the vaccine hesitancy literature, in particular the WHO “Three C's” model and the vaccine hesitancy determinants matrix [26], as well as the UK SAGE report on vaccine hesitancy in ethnic minority groups [15], Selected variables measured trust in vaccines and those delivering them; perceived risk of COVID-19; access to vaccines based on job role, sector and location; socio-demographics; and psychological factors. See Supplementary Table 1 for variable list and the data dictionary for details of variables https://www.uk-reach.org/main/data-dictionary/. Importantly, we classified ethnicity by using the Office for National Statistics 18 ethnicity categories to ensure we aligned with national data and statistics (see Supplementary table 1 for details of categories). Ethnicity is a complex construct; it has been defined as “the social group a person belongs to, and either identifies with or is identified with by others, as a result of a mix of cultural and other factors including language, diet, religion, ancestry and physical features traditionally associated with race” [37]. Religion was included to determine its effect on hesitancy and control for any potential confounding with ethnicity.

We included a variable to indicate whether participants had answered VQ1 (between 4^th^ and 20^th^ December 2020) or VQ2 (between 21^st^ December 2020 and 19^th^ February 2021). In addition, participants whose VQ2 response indicated that they had considered or were considering not having the vaccine were asked to indicate why they were hesitant.

#### 2.2.4 Statistical analysis

We summarised categorical variables as count and percentage, and continuous variables as mean (standard deviation [SD]) or median (interquartile range [IQR]) depending on their distribution. We compared groups (hesitant vs accepting, and ethnic groups) with chi-squared tests for categorical variables, and t-tests and analyses of variance for continuous measures, with non-parametric equivalents used as appropriate. Due to the number of tests being performed we considered associations statistically significant at p≤0•001. We checked 2 way interactions of ethnicity with other significant predictors of hesitancy using complete cases, and used a likelihood ratio test to determine whether there was an improvement in model fit by inclusion of the interaction term.

We used univariable and multivariable logistic regression to determine unadjusted and adjusted associations of variables described above with SARS-CoV-2 vaccine hesitancy.

We used multiple imputation (MI) to replace missing data in all logistic regression models using the package *mice* (Multiple Imputation by Chained Equations) v3·13·0 in *R* version 4·0·4, using predictive mean matching (pmm) for all variables, with 20 imputations and five iterations per imputation (See Supplementary Table 2).

### 2.3 Qualitative data and analysis

#### 2.3.1 Study population

A purposive sample of clinical and non-clinical HCWs aged 16 or older from ethnic minority and White backgrounds with experience of working in UK healthcare settings during COVID-19, recruited through study partners, community organisations, and NHS organisations across the UK.

#### 2.3.2 Data collection and analysis

Data were collected through in-depth semi-structured interviews and focus groups using a piloted topic guide. The topic guide explored experiences of working during the COVID-19 pandemic, fears and concerns, stigma, discrimination, racism, views on the COVID-19 vaccine, challenges participants encountered in accessing information, and their perceived risk. Interviews and focus groups were recorded and transcribed prior to analysis. We also collected data on participant gender, ethnicity, age, country of birth, and job role. Free text data collected through the cohort study in response to the following three questions were also included: “What are your thoughts on why people from ethnic minorities working in health and care have been more severely affected by COVID-19?”, “How do you see society changing as a result of COVID-19?” and “How do you see your own future changing as a result of COVID-19?” [38]

We used framework analysis to analyse anonymised transcripts from interviews and focus groups, and free text data from the cohort study. We developed the initial framework based on a preliminary thematic analysis of the data and the WHO framework for behavioural considerations for acceptance and uptake of COVID-19 vaccines [39]. The framework encompasses “Drivers of vaccine hesitancy” relating to “health information and messaging”, “Motivation” utilising the ‘Three Cs Model’, and “Improving delivery”. We piloted the framework with the first five transcripts, and refined it iteratively during analysis. Throughout analysis, the framework, and new codes and themes were discussed by all researchers to achieve a consensus in the analysis, and to strengthen consistency, transparency, and trustworthiness. Throughout the analysis, the researchers engaged in an active process of reflexivity.

### 2.4 Ethical approval

Both studies were approved by the Health Research Authority (Brighton and Sussex Research Ethics Committee; ethics reference: 20/HRA/4718). All participants gave written informed consent.

### 2.5 Involvement and engagement

We worked closely with a Professional Expert Panel of HCWs from a range of ethnic backgrounds, occupations, and genders, as well as with national and local organisations (see study protocols) [33,34].

### 2.6 Role of the funding source

The funders had no role in study design, data collection, data analysis, interpretation, writing of the report

## 3 Results

### 3.1 Prospective nationwide cohort study

#### 3.1.1 Description of analysed cohort

Between 4^th^ December 2020 and 19^th^ February 2021, professional regulators sent 1,052,875 HCWs email invitations with a link to the study website, and 21 National Health Service (NHS) Hospital Trusts publicised the questionnaire to their staff and invited staff by email. Approximately 46% of the emails (480,111) sent by the regulators were received/open. As of 19^th^ February 2021, 24,601 participants had clicked through to the study website and registered.

As of 19^th^ February 2021, 15,151 participants had started the questionnaire. The analysed interim cohort were formed of 11,584 participants who both completed the questionnaire and answered the question about their sex. This gives an effective response rate of 47.1% of those who registered on the study website (and 76.5% of those who started the questionnaire, 1.1% of those who were sent an email and 3.5% of those who opened the email). See Table 1 for the cohort demographics, occupation and location. Supplementary Table 2 provides an estimate of potential bias.

**Table 1:** Demographic and occupational characteristics of cohort.

Variable	Total(*n*=11,584)
**Age, median (IQR)**	45 (34 - 54)
**Sex, n(%)**	
Male	2797 (24·2%)
Female	8787 (75·9%)
**Ethnicity, n(%)**	
White - English, Welsh, Scottish, Northern Irish	6907 (60·8%)
White - Irish	209 (1·8%)
White - Other/Gypsy Irish Traveller	878 (7·7%)
Asian - Indian	1187 (10·4%)
Asian - Pakistani	315 (2·8%)
Asian - Bangladeshi	69 (0·6%)
Asian - Chinese	253 (2·2%)
Asian - Other	365 (3·2%)
Black - African	349 (3·1%)
Black - Caribbean	102 (0·9%)
Black - Other	20 (0·2%)
Mixed - White & Black African	66 (0·6%)
Mixed - White & Black Caribbean	84 (0·7%)
Mixed - White & Asian	179 (1·6%)
Mixed - Other	142 (1·3%)
Other - Arab	122 (1·1%)
Other	123 (1·1%)
**Religion, n(%)**	
None	3939 (36·0%)
Christian	5109 (46·7%)
Buddhist	133 (1·2%)
Hindu	697 (6·4%)
Jewish	107 (1·0%)
Muslim	670 (6·1%)
Sikh	120 (1·1%)
Other	156 (1·4%)
**Country of birth, n(%)**	
UK	8335 (73·4%)
Outside UK	3024 (26·6%)
**Job role, n(%)**	
Doctors and medical support	2679 (24·0%)
Nurses, NAs, Midwives	2300 (20·6%)
Allied Health Professionals*	4959 (44·5%)
Dental	716 (6·4%)
Administrative/Estates/Other	488 (4·4%)
**Job location, n(%)**	
Not in hospital	5013 (44·5%)
Hospital	6254 (55·5%)
**IMD quintile**	
1 (most deprived)	965 (9·5%)
2	1660 (16·4%)
3	2097 (20·7%)
4	2478 (24·5%)
5 (least deprived)	2926 (28·9%)

⁎ Also includes pharmacists, healthcare scientists, ambulance workers and those in optical roles.

#### 3.1.2 Univariable results

Table 2 shows the cohort stratified by SARS-CoV-2 vaccine hesitancy; Table 3 shows univariable relationships between hesitancy and vaccine-related trust and perceived risk of COVID-19. Briefly, just under a quarter of participants (2694/11,584; 23·3%) were vaccine hesitant. Over half (51·0%) of Black Caribbean, 38·1% of Mixed White and Black Caribbean, 34·4% of Black African, 32·4% of Chinese, 29·8% of Pakistani, and 28·7% of the White Other^1^ group were vaccine hesitant, compared to 21·0% of White British, 19·6% of Indian and 18·8% of Bangladeshi HCWs. The least hesitant occupational group was the Doctors and medical support group (18·4% hesitant) and the most hesitant was the Nursing, Nursing associates and Midwives group (28·2% hesitant).

**Table 2:** Demographic characteristics of cohort stratified by SARS-CoV-2 vaccine hesitancy.

Variable	SARS-CoV-2 vaccine		
	Not hesitant 8691 (75•0%)	Hesitant 2694 (23•3%)	Missing 199 (1•7%)
**Age, median (IQR)**	46 (36 - 55)	40 (31 - 51)	43 (34 - 54)
**Sex, n(%)**			
Male	2298 (82.2%)	466 (16.7%)	33 (1.2%)
Female	6393 (72.8%)	2228 (25.4%)	166 (1.9%)
**Ethnicity, n(%)**			
White - English, Welsh, Scottish, Northern Irish	5365 (77.7%)	1452 (21.0%)	90 (1.3%)
White - Irish	156 (74.6%)	50 (23.9%)	<5 (<2%)
White - Other/Gypsy Irish Traveller	606 (69.0%)	252 (28.7%)	20 (2.3%)
Asian - Indian	936 (78.9%)	232 (19.6%)	19 (1.6%)
Asian - Pakistani	215 (68.3%)	94 (29.8%)	6 (1.9%)
Asian - Bangladeshi	55 (79.7%)	13 (18.8%)	<5 (<2%)
Asian - Chinese	166 (65.6%)	82 (32.4%)	5 (2•0%)
Asian - Other	269 (73.7%)	89 (24.4%)	7 (1.9%)
Black - African	210 (60.2%)	120 (34.4%)	19 (5.4%)
Black - Caribbean	44 (43.1%)	52 (51.0%)	6 (5.9%)
Black - Other	11 (55.0%)	7 (35.0%)	<5 (<2%)
Mixed - White & Black African	47 (71.2%)	18 (27.3%)	<5 (<2%)
Mixed - White & Black Caribbean	50 (59.5%)	32 (38.1%)	<5 (<2%)
Mixed - White & Asian	140 (78.2%)	37 (20.7%)	<5 (<2%)
Mixed - Other	103 (72.5%)	36 (25.4%)	<5 (<2%)
Other - Arab	83 (68.0%)	34 (27.9%)	5 (4.1%)
Other	89 (72.4%)	33 (26.8%)	<5 (<2%)
**Religion, n(%)**			
None	2995 (76.0%)	896 (22.8%)	48 (1.2%)
Christian	3827 (74.9%)	1176 (23.0%)	106 (2.1%)
Buddhist	93 (69.9%)	36 (27.1%)	<5 (<2%)
Hindu	557 (79.9%)	132 (18.9%)	8 (1.2%)
Jewish	93 (86.9%)	13 (12.2%)	<5 (<2%)
Muslim	472 (70.5%)	184 (27.5%)	14 (2.1%)
Sikh	89 (74.2%)	28 (23.3%)	<5 (<2%)
Other	99 (63.5%)	55 (35.3%)	<5 (<2%)
**Country of birth, n(%)**			
UK	6411 (75.8%)	1924 (22.7%)	127 (1.5%)
Outside UK	2264 (73.1%)	760 (24.6%)	72 (2.3%)
**Job role, n(%)**			
Doctors and medical support	2166 (80.9%)	493 (18.4%)	20 (0.8%)
Nurses, NAs, Midwives	1598 (69.5%)	648 (28.2%)	54 (2.4%)
Allied Health Professionals*	3689 (74.4%)	1184 (23.9%)	86 (1.7%)
Dental	551 (77.0%)	151 (21.1%)	14 (2.0%)
Administrative/Estates/Other	369 (75.6%)	106 (21.7%)	13 (2.7%)
**Job location, n(%)**			
Not in hospital	3859 (77.0%)	1072 (21.4%)	82 (1.6%)
Hospital	4595 (73.5%)	1555 (24.9%)	104 (1.7%)
**IMD quintile**			
1 (most deprived)	661 (68.5%)	281 (29.1%)	23 (2.4%)
2	1169 (70.4%)	458 (27.6%)	33 (2.0%)
3	1574 (75.1%)	479 (22.8%)	44 (2.1%)
4	1900 (76.7%)	527 (21.3%)	51 (2.1%)
5 (least deprived)	2294 (78.4%)	603 (20.6%)	29 (1.0%)

⁎ Also includes pharmacists, healthcare scientists, ambulance workers and those in optical roles

**Table 3:** Selected predictor variables stratified by SARS-CoV-2 vaccine hesitancy.

Variable	Total(n=11,584)	SARS-CoV-2 vaccine			*P* value
		Not hesitant 8691 (75•0%)	Hesitant 2696 (23•3%)	Missing 199 (1•7%)	
**TRUST VARIABLES**					
**Belief in COVID-19 ‘conspiracies’ score, med (IQR)** (min 6 [does not believe] - max 24 [strongly believes])	9 (8 - 10)	8 (7 - 10)	10 (8 - 11)	10 (9 - 13)	<0·0001*
**Pro-vaccine score, med (IQR)** (min 4 [anti-vaccination] - max 20 [pro-vaccination])	16 (14 - 17)	16 (14 - 17)	14 (12 - 16)	13 (11 - 16)	<0·0001*
**Influenza vaccination status 2019 - 2020, n(%)**					
Vaccinated	8279 (71·9%)	6569 (76·0%)	1605 (59·9%)	105 (54·1%)	<0·0001*
Unvaccinated	3233 (28·1%)	2070 (24·0%)	1074 (40·1%)	89 (45·9%)	
**Trust in employer to address a concern about unsafe clinical practice, n(%)**					
1 (does not trust employer)	356 (3·3%)	252 (3·1%)	95 (3·8%)	9 (5·1%)	<0·0001†
2	950 (8·9%)	649 (8·1%)	289 (11·6%)	12 (6·8%)	
3	1807 (16·9%)	1241 (15·4%)	532 (21·3%)	34 (19·2%)	
4	4113 (38·4%)	3113 (38·7%)	942 (37·8%)	58 (32·8%)	
5 (trusts employer)	3481 (32·5%)	2781 (34·6%)	636 (25·5%)	64 (36·2%)	
**Discrimination at work on the basis of ethnicity, nationality or religion, n(%)**					
Has not experienced discrimination	9270 (86·6%)	7072 (87·9%)	2063 (83·1%)	135 (78·5%)	<0·0001*
Has experienced discrimination	1434 (13·4%)	977 (12·1%)	420 (16·9%)	37 (21·5%)	
**RISK VARIABLES**					
**Previous laboratory evidence of COVID-19 (PCR or serology), n(%)**					
Never tested	1903 (16·5%)	1449 (16·6%)	420 (15·6%)	34 (17·4%)	<0·0001*
Tested negative	7350 (63·5%)	5635 (64·9%)	1610 (59·8%)	105 (53·6%)	
Tested positive	2316 (20·0%)	1597 (18·4%)	662 (24·6%)	57 (29·1%)	
**Number of comorbidities, n(%)**					
0	7841 (70·7%)	5801 (69·6%)	1910 (74·1%)	130 (73·5%)	<0·0001*
1	2528 (22·8%)	1956 (23·5%)	532 (20·6%)	40 (22·6%)	
≥2	724 (6·5%)	580 (7·0%)	137 (5·3%)	7 (4·0%)	
**Pregnancy, n(%)**					
Not pregnant	10,948 (98·7%)	8286 (99·4%)	2490 (96·7%)	194 (97·5%)	<0·0001*
Pregnant	141 (1·3%)	50 (0·6%)	86 (3·3%)	5 (2·5%)	
**Perceived risk of hospitalisation with COVID-19, med, (IQR)**					
(100 point scale)	20 (5 - 50)	20 (5 - 50)	20 (5 - 40)	15 (3 - 50)	0·0004†
**Concerned about unknowingly spreading COVID-19, n(%)**					
Not concerned	5745 (49·8%)	4285 (49·5%)	1362 (50·8%)	98 (50·8%)	0·2*
Concerned	5781 (50·2%)	4367 (50·5%)	1319 (49·2%)	95 (49·2%)	
**Exposed to COVID-19 patients at work, n(%)**					
Unexposed	7164 (66·1%)	5476 (67·3%)	1589 (62·9%)	99 (55·3%)	<0·0001*
Exposed	3682 (34·0%)	2663 (32·7%)	939 (37·1%)	80 (44·7%)	

⁎ chi-square,

† Wilcoxon rank-sum for comparison between hesitant and non-hesitant cohorts. For categorical variables, percentages are column wise apart from totals which are computed row wise. For details of the derivation of the trust variables please see supplementary information. Comorbidities Include: organ transplant, diabetes, heart disease, hypertension, stroke, kidney disease, liver disease, anaemia, asthma, lung disease, cancer, neurological disorder and immunosuppression

Hesitant participants scored higher on the ‘COVID-19 conspiracy beliefs’ scale (median hesitant: 10, IQR: 8-11; non-hesitant: 8, 7-10 p<0·0001), were less confident their employer would address a concern about unsafe clinical practice (63·3% vs 73·3% p<0·001) and were more likely to have laboratory evidence of previous SARS-CoV-2 infection (24·6% vs 18·4%, p<0·001) compared to the non-hesitant cohort. 136 pregnant HCWs were included in the analysis, of whom 86 (63·2%) were SARS-CoV-2 vaccine hesitant. Supplementary Table 4 shows vaccine-related trust and risk factors stratified by ethnicity.

*Reasons for hesitancy*: Among those who reported reasons for SARS-CoV-2 vaccine hesitancy, the White group (White British, White Irish, White Other and White Gypsy/Irish Traveller) were less concerned than other ethnic groups about potential vaccine side effects or about the vaccine not having been tested in diverse ethnic groups, and they were less likely to want to delay until others had the vaccine. Reasons for hesitancy overall and by broad ethnic grouping are given in Supplementary Table 5.

#### 3.1.3 Multivariable results

*Demographic predictors of SARS-CoV-2 vaccine hesitancy*: Table 4 shows univariable and multivariable logistic regression models with an outcome of SARS-CoV-2 vaccine hesitancy. After adjusting for socio-demographic, job, trust, perceived COVID-19 risk, and psychological factors, vaccine hesitancy was less likely with increasing age (aOR 0·74 95%CI 0·70–0·78 for each decade increase) and more likely among female HCWs (aOR 1·42 95%CI 1·24–1·62). Compared to White British HCWs, those from Black Caribbean (aOR 3·37 95%CI 2·11–5·37), Black African (aOR 2·05, 95%CI 1·49–2·82), and White Other (aOR 1·48 95%CI 1·19–1·84) ethnic groups were significantly more likely to be vaccine hesitant. Results remained broadly unchanged using a reduced five-category ethnicity classification (White, Asian, Black, Mixed and Other).

**Table 4:** Unadjusted and adjusted analysis of SARS-CoV-2 vaccine hesitancy predictors.

Variable	OR (95% CI)	p value	aOR (95% CI)	p value
**Age** (for each decade increase)	0.71 (0.69 - 0.74)	<0.001	0.74 (0.70 - 0.78)	<0.001
**Sex**				
Male	Ref	-	Ref	-
Female	1.72 (1.54 - 1.93)	<0.001	1.42 (1.24 - 1.62)	<0.001
**Ethnicity**				
White - British	Ref	-	Ref	-
White - Irish	1.18 (0.85 - 1.63)	0.32	1.39 (0.96 - 2.02)	0.08
White - Other/Gypsy Irish Traveller	1.55 (1.33 - 1.81)	<0.001	1.48 (1.19 - 1.84)	0.001
Asian - Indian	0.92 (0.79 - 1.07)	0.28	0.76 (0.57 - 1.02)	0.07
Asian - Pakistani	1.62 (1.26 - 2.08)	<0.001	1.18 (0.78 - 1.79)	0.42
Asian - Bangladeshi	0.87 (0.47 - 1.59)	0.64	0.66 (0.32 - 1.39)	0.28
Asian - Chinese	1.80 (1.37 - 2.36)	<0.001	1.59 (1.15 - 2.20)	0.005
Asian - Other	1.23 (0.96 - 1.57)	0.1	1.03 (0.74 - 1.42)	0.86
Black - African	2.09 (1.66 - 2.63)	<0.001	2.05 (1.49 - 2.82)	<0.001
Black - Caribbean	3.91 (2.62 - 5.84)	<0.001	3.37 (2.11 - 5.37)	<0.001
Black - Other	2.45 (0.99 - 6.06)	0.05	1.63 (0.52 - 5.06)	0.40
Mixed - White & Black Caribbean	2.23 (1.43 - 3.48)	<0.001	1.62 (0.98 - 2.67)	0.06
Mixed - White & Black African	1.35 (0.78 - 2.33)	0.28	1.36 (0.87 - 2.11)	0.33
Mixed - White & Asian	0.95 (0.66 - 1.38)	0.79	0.89 (0.59 - 1.36)	0.60
Mixed - Other	1.29 (0.88 - 1.90)	0.19	1.35 (0.87 - 2.11)	0.18
Arab	1.43 (0.96 - 2.13)	0.08	1.65 (0.97 - 2.82)	0.07
Other	1.36 (0.91 - 2.03)	0.13	1.41 (0.88 - 2.26)	0.15
**Job role**				
Doctors and medical support	Ref	-	Ref	-
Nurses, NAs, Midwives	1.75 (1.54 - 2.00)	<0.001	1.17 (0.98 - 1.41)	0.08
Allied Health Professionals*	1.39 (1.24 - 1.57)	<0.001	0.99 (0.85 - 1.16)	0.90
Dental	1.21 (0.99 - 1.48)	0.06	0.75 (0.58 - 0.97)	0.03
Admin / estates / other	1.25 (0.99 - 1.57)	0.06	1.03 (0.78 - 1.36)	0.86
**Job location**				
Not in hospital	Ref	-	Ref	-
Hospital	1.22 (1.12 - 1.34)	<0.001	1.18 (1.06 - 1.32)	0.004
**Religion**				
No religion	Ref	-	Ref	-
Christian	1.03 (0.94 - 1.14)	0.52	0.99 (0.87 - 1.12)	0.85
Buddhist	1.27 (0.86 - 1.86)	0.23	1.10 (0.70 - 1.72)	0.68
Hindu	0.83 (0.68 - 1.02)	0.08	1.10 (0.79 - 1.53)	0.58
Jewish	0.49 (0.27 - 0.88)	0.02	0.54 (0.28 - 1.03)	0.06
Muslim	1.31 (1.09 - 1.58)	0.003	1.02 (0.73 - 1.42)	0.92
Sikh	1.10 (0.72 - 1.68)	0.67	1.39 (0.81 - 2.38)	0.24
Other	1.74 (1.25 - 2.44)	0.001	1.77 (1.19 - 2.62)	0.005
**Religiosity**	1.10 (1.05 - 1.15)	<0.001	1.03 (0.97 - 1.10)	0.38
**Country of Birth**				
Outside UK	Ref	-	Ref	-
UK	0.89 (0.80 - 0.97)	0.01	1.26 (1.07 - 1.48)	0.006
**IMD quintile**				
1 (most deprived)	1.38 (1.16 - 1.64)	<0.001	0.96 (0.79 - 1.17)	0.66
2	1.26 (1.08 - 1.46)	0.003	1.10 (0.93 - 1.31)	0.26
3	Ref	-	Ref	-
4	0.93 (0.81 - 1.07)	0.32	1.01 (0.86 - 1.18)	0.90
5 (least deprived)	0.87 (0.76 - 0.99)	0.04	1.00 (0.87 - 1.16)	0.95
**Influenza vaccination 2019 - 2020**				
Unvaccinated	Ref	-	Ref	-
Vaccinated	0.46 (0.43 - 0.51)	<0.001	0.51 (0.46 - 0.57)	<0.001
**Pro-vaccine attitudes**	0.78 (0.73 - 0.84)	<0.001	0.82 (0.78 - 0.86)	<0.001
**COVID-19 conspiracy beliefs scale**	1.22 (1.20 - 1.24)	<0.001	1.12 (1.08 - 1.16)	<0.001
**Trust in employer:**				
Feel secure raising concerns about unsafe clinical practice	0.85 (0.81 - 0.88)	<0.001	1.02 (0.96 - 1.09)	0.51
Feel confident concerns would be addressed	0.82 (0.78 - 0.85)	<0.001	0.87 (0.82 - 0.93)	<0.001
**Discrimination at work** on the basis of ethnicity, nationality or religion	1.45 (1.28 - 1.64)	<0.001	0.99 (0.84 - 1.17)	0.93
**Number of comorbidities**				
0	Ref	-	Ref	-
1	0.83 (0.75 - 0.93)	0.001	0.97 (0.85 - 1.10)	0.65
≥2	0.72 (0.59 - 0.87)	0.001	1.11 (0.87 - 1.40)	0.40
**BMI category**				
<18.5	1.04 (0.72 - 1.52)	0.81	0.89 (0.58 - 1.36)	0.58
18.5 to <25	Ref	-	Ref	-
25 to <30	0.91 (0.82 - 1.01)	0.07	0.90 (0.80 - 1.02)	0.09
30 to <40	0.96 (0.85 - 1.09)	0.52	0.87 (0.76 - 1.01)	0.07
≥ 40	0.87 (0.65 - 1.16)	0.33	0.68 (0.50 - 0.95)	0.02
**Pregnancy**				
Not pregnant	Ref	-	Ref	-
Pregnant	5.87 (4.14 - 8.33)	<0.001	7.12 (4.74 - 10.70)	<0.001
**Previous evidence of COVID-19 (PCR or serology)**				
Negative	Ref	-	Ref	-
Never tested	1.02 (0.90 - 1.15)	0.76	0.95 (0.83 - 1.10)	0.52
Positive	1.46 (1.32 - 1.62)	<0.001	1.30 (1.14 - 1.47)	<0.001
**Perceived risk of hospitalisation with COVID-19** (for each 10 point increase)	0.97 (0.95 - 0.99)	0.001	0.97 (0.94 - 0.99)	0.009
**Perceived risk of unknowingly spreading COVID-19**				
Not concerned	Ref	-	Ref	-
Quite or very concerned	0.95 (0.87 - 1.03)	0.22	0.88 (0.79 - 0.97)	0.01
**Exposure to COVID-19 patients at work**				
Unexposed	Ref	-	Ref	-
Exposed	1.22 (1.11 - 1.34)	<0.001	0.90 (0.80 - 1.01)	0.07
**Personality factors**				
Agreeableness	0.97 (0.96 - 0.99)	0.001	0.98 (0.96 - 0.99)	0.01
Conscientiousness	0.99 (0.98 - 1.01)	0.40	1.00 (0.98 - 1.02)	0.75
Extraversion	0.98 (0.97 - 0.99)	0.003	0.99 (0.98 - 1.01)	0.04
Neuroticism	1.02 (1.01 - 1.04)	<0.001	0.99 (0.98 - 1.00)	0.44
Openness	0.99 (0.97 - 1.00)	0.03	0.99 (0.98 - 1.01)	0.23
**Fatalism**	1.03 (1.02 - 1.04)	<0.001	1.00 (0.99 - 1.01)	0.54
**Information sources**				
Friends	1.40 (1.19 - 1.65)	<0.001	1.27 (1.02 - 1.57)	0.03
Mainstream media	0.68 (0.60 - 0.76)	<0.001	0.82 (0.70 - 0.96)	0.01
Official	0.77 (0.63 - 0.94)	0.01	0.85 (0.66 - 1.10)	0.21
Scientific	0.88 (0.76 - 1.02)	0.09	1.28 (1.07 - 1.53)	0.007
**Time of questionnaire completion**				
December 2020	Ref	-	Ref	-
January 2021 or after	0.49 (0.45 - 0.54)	<0.001	0.52 (0.30 - 0.90)	<0.001

⁎ Also includes pharmacists, healthcare scientists, ambulance workers and those in optical roles

*Trust, COVID-19 risk and psychological predictors of SARS-CoV-2 vaccine hesitancy and refusal*: Greater belief in COVID-19 conspiracies was significantly associated with increased odds of hesitancy (aOR 1·12, 95%CI 1·08–1·16 for each 1 point increase on the scale). Increasing confidence that concerns raised about unsafe practice would be addressed by their employer decreased the odds of hesitancy (aOR 0·87, 95%CI 0·82 - 0·93). Those who had received the influenza vaccine in winter 2019/2020 were around half as likely to be SARS-CoV-2 vaccine hesitant compared to those who had not (aOR 0·51, 95%CI 0·46 - 0·57). HCWs who reported testing positive for SARS-CoV-2 by PCR or serology, were significantly more likely to be hesitant than those testing negative (aOR 1·30, 95%CI 1·14 - 1·47). Pregnant HCWs were over 7 times as likely to be hesitant (aOR 7·12, 95%CI 4·74 - 10·70). 2 way interaction terms between ethnicity and other significant predictors did not improve model fit.

Significant predictors of vaccine hesitancy on multivariable analysis remained unchanged when we coded participants who had accepted the vaccine as non-hesitant (even if they had considered not having the vaccine)(data not shown).

### 3.2 Qualitative study

#### 3.2.1 Description of sample

We included 99 individuals, 41 recruited through interviews (n=24) and focus groups (n=17), and 58 from the longitudinal cohort study (free text comments provided about vaccinations). Among the 41 qualitative participants, 13 were Asian (32%), 12 were Black (29%), and 10 were White (24%). 27 (66%) were women, and 24 were born in the UK (59%). 18 participants were allied health professionals, pharmacists, and dentists (44%), whilst 9 were doctors (22%), 3 were nurses or midwives (7%), and 11 were non-clinical (27%). Among the 58 cohort participants, 42 were White (72%), 8 were Asian (14%), and 4 were Black (7%). 48 participants (83%) were women, and 44 (76%) were born in the UK. 26 (45%) of participants were allied health professionals, pharmacists, or dentists, 7 were doctors (12%), whilst 23 (40%) were nurses or midwives.

#### 3.2.2 Drivers of vaccine hesitancy

**Fig. 1:**
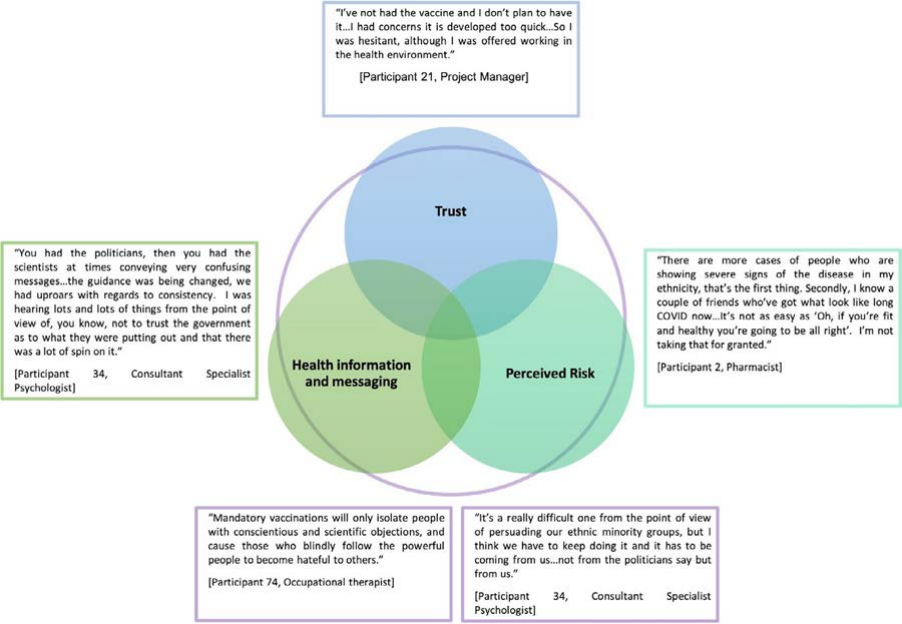
Qualitative themes.

We identified four intersecting themes describing key drivers of and ways to address vaccine hesitancy among HCWs: Trust, Perceived risk, Health information and messaging, and Improving delivery (See Fig. 1; Supplementary Tables 6 – 9 for quotes).

*Trust*: Participants described their enthusiasm about the vaccine, appreciation of being prioritised, and the role of trust in colleagues, the NHS, and health information in facilitating vaccine uptake. Narratives also highlighted the influence of experiences of discrimination and structural inequities on trust and vaccine hesitancy, and the ubiquity of concerns around the vaccine across both those who declined to be vaccinated and those who described themselves as pro-vaccine.

*Trust in vaccinations*: Whilst some participants described a lack of confidence in vaccines generally, most participants described being accepting of routine or flu vaccinations. Key concerns for the COVID-19 vaccine related to speed of development, lack of longitudinal data, and potential side effects, as well as efficacy against SARS-CoV-2 variants. There were also concerns about the underrepresentation of individuals from ethnic minority backgrounds in vaccine trials.

*Trust in those producing, giving, and taking vaccines*: Vaccine confidence among colleagues, family, friends and community members increased HCW trust in the vaccine. More senior colleagues - particularly clinicians - were especially influential, and conversely trust was eroded when they did not adhere to guidance. Some participants described a contradiction between their own concerns around having the vaccine, yet promoting it for the wider public through their roles.

*Perceived risks of COVID-19 to self and others*: Whilst some participants felt at low risk, others expressed concern about the risk of exposure in their role and fears of having COVID-19, even if they did not have other key risk factors. Previous infection, knowing people who had been unwell or passed away from COVID-19, and concerns about infection of their families and loved ones often increased perceived risk. Participants’ views about the extent to which vaccination could reduce risk also influenced their decision to be vaccinated, as did their desire to reduce their risk of transmission and protect their close contacts. Participants also discussed how they perceived ethnicity to influence risk. Whilst prioritisation of NHS workers for vaccines was welcomed, some felt ethnic minority groups should have been prioritised given their increased risk.

*Health information and messaging*: Accessibility and trustworthiness of health information shaped vaccine concerns. Complex information, conflicting and changing guidance, overwhelming amounts of material, and poor provision of information in other languages contributed to a lack of trust, confusion, and ultimately vaccine hesitancy. Participants also noted the stigma around vaccine hesitancy and lack of vaccine knowledge.

Participants obtained information from numerous sources. Social media was often described as potentially misleading or unreliable, but some participants acknowledged its usefulness for raising awareness, vaccine promotion, and disseminating messaging, especially as information shared by community members may be more trusted. Participants also frequently accessed information through the news, or Government and NHS sources. However, the positive presentation of vaccines by these sources was felt by some to be insincere with potential risks not being transparently communicated. This fed into suspicions around official reports on COVID-19 further contributing to HCW mistrust.

There were varying responses to the focus on ethnic minorities. While prioritisation of NHS workers for vaccines was welcomed, some felt HCWs from ethnic minority backgrounds should have been further prioritised given evidence of the disproportionate impact of COVID-19 on these communities. The narratives also illustrated discomfort with the focus on ethnic minorities in the media, messaging and vaccine promotion campaigns, which singled out these communities as ‘vaccine hesitant’ and increased stigma. One participant brought attention to discourse around reported low vaccine uptake of the vaccine among Black doctors, calling for greater transparency and accuracy around uptake rates, and better understandings of the factors that inform decisions about vaccines.

*Inclusive communication*: Participants highlighted the value of communicating messages through a range of media and languages, and engaging directly with people to respond to questions or concerns, and tackle misinformation. Participants also advocated for using existing resources such as multilinguistic healthcare staff to strengthen the accessibility and trustworthiness of health information.

Participants also described the importance of language in how groups are described, and the need to avoid assumptions or stereotyping associated with ethnicity. This was important for creating more inclusive communication around how at-risk groups - and ethnic minority communities in particular - are described in research, the workplace, and the media.

*Increasing transparency and trust*: Trust and informed decision-making about vaccines was influenced by how risk groups were identified and prioritised, who was eligible, and the perceived risks and benefits. Participants explained the importance of transparent and clear communication through hospital Trusts.

*Equity, opportunity and mandatory vaccination*: Participants underscored the need to ensure equity in vaccine delivery, with some advocating prioritisation of staff experiencing the greatest barriers to getting the vaccine, or who were at greatest risk. Whilst some participants advocated *for “mandatory vaccinations for those choosing to work in health and social care settings”* (Participant 84, Speech and language therapist), others were concerned about the potential lack of equity for those who chose not to have the vaccine, and that mandating vaccination could create further ethnic divides between communities and increase stigma and discrimination. Participants also discussed how ensuring equity in accessibility and opportunity to have the vaccine would be paramount for improving delivery.

*Outreach through involvement*: Participants described how the vaccine roll-out could be improved through better engagement with and involvement of HCWs, particularly those from ethnic minority communities. The narratives pointed to the lack of inclusion of marginalised communities throughout the pandemic, and the potential benefit of increasing visibility of less well represented groups in the media to promote vaccine uptake and trust.

Participants also discussed the importance of promoting vaccination through trusted networks, and the value of more proactive involvement and engagement of healthcare workers from diverse ethnic backgrounds. An important aspect of both building trust and increasing accessibility was acknowledging cultural differences in understandings of and access to vaccines. Participants highlighted how the involvement of minoritised communities can play an important role in bridging cultural divides, and the potential benefit of outreach activities for addressing logistical challenges in delivering the vaccine.

## 4 Discussion

In this analysis of interim data from nearly 12,000 HCWs across the UK, approximately a quarter of participants reported SARS-CoV-2 vaccine hesitancy. HCWs from Black Caribbean, Black African and White Other ethnic groups reported higher hesitancy than those from the White British group after adjusting for other predictors. Additional factors predicting hesitancy were scoring higher on the COVID-19 conspiracy beliefs scale, lower trust in employer, pregnancy, and previous COVID-19. Qualitative data showed information and messaging influenced vaccine concerns. Speed of vaccine development, experiences of discrimination and structural inequalities also contributed to a lack of trust in the vaccine.

Our finding of 23% of UK HCWs being SARS-CoV-2 vaccine hesitant is in keeping with a recent systematic review of COVID-19 vaccine uptake, which found an average acceptance of 57% (range 28%-78%) across countries and occupation groups [8]. Many smaller SARS-CoV-2 vaccine hesitancy studies have been conducted outside the UK with common predictors of vaccine hesitancy being female sex, non-medical occupation, lack of influenza vaccination and lower perceived risk of COVID-19 [6,7,[40], [41], [42]]. Vaccine hesitancy amongst female HCWs, in particular, is an important finding given that females make up a significant proportion of the UK healthcare workforce and further work to understand this is urgently required. There is conflicting evidence regarding the effect of age and previous COVID-19 on SARS-CoV-2 vaccine hesitancy [40,[43], [44], [45]]. Importantly, only two studies, both conducted in the US, examined the impact of ethnicity on SARS-CoV-2 vaccine hesitancy after adjustment for confounders, with both finding that Black ethnic groups were more likely to be hesitant compared to White HCWs [44,45]. Whilst data on vaccine hesitancy in UK HCWs are limited, recent work examining vaccine uptake amongst hospital staff in the UK found that 35·5% of HCWs had not been vaccinated; vaccination rates were highest amongst White HCWs and, in-line with our findings, lowest among Black ethnic groups [12].

Due to the novel nature of COVID-19, the evidence base for barriers to SARS-CoV-2 vaccination in ethnic minority communities is limited. However, UK's SAGE ethnicity subgroup identified barriers to vaccine uptake amongst ethnic minority groups including lower trust in vaccine efficacy/safety (particularly speed of vaccine development), mistrust of healthcare organisations (due to prior unethical research practices), lack of representation in vaccine trials, and institutional racism and discrimination [30]. Our study provides evidence that these same factors may influence vaccine hesitancy in HCWs. Many of these themes emerged in our qualitative data, with HCWs describing reservations about accepting vaccination rooted in safety concerns due to the short development timeframe of current SARS-CoV-2 vaccines. Experiences of health inequities and knowledge of historic unethical health and research practices were cited by some Black HCWs as influencing their mistrust of the NHS. This overarching mistrust in the organisation was also reflected in attitudes towards SARS-CoV-2 vaccination with a perception of low ethnic minority involvement in trials to gauge vaccine safety/efficacy, and the lack of prioritisation within the vaccination rollout despite evidence of the disproportionate impact on the health of those from minority ethnic backgrounds. Additionally, in data from the cohort study, lower trust in one's employer was found to predict hesitancy, and high proportions of vaccine hesitant ethnic minority HCWs expressed concerns regarding vaccine safety and about a lack of testing in all ethnic groups.

These results have important implications for public health measures aimed at improving vaccine uptake. It has been reported that mandatory SARS-CoV-2 vaccination is being considered for care home staff in the UK [46], and the Italian government has mandated vaccination in HCWs (with those that refuse being offered duties that do not risk viral transmission or suspension without pay) [47]. Whilst these measures may improve vaccine uptake, our results indicate that implementing these policies may undermine trust (both in the employing healthcare organisations and in the vaccination programme) [48]. Given that this effect would not be seen equally across ethnic groups, such interventions have the potential to increase stigma and discrimination and widen ethnic disparities.

We found that higher scores on the COVID-19 conspiracy beliefs scale was associated with vaccine hesitancy, and this was also more likely in ethnic minority groups as compared to those of White ethnicity. To our knowledge, we are the first to show this effect in a HCW population. A general population survey in the UK found that belief in COVID-19 conspiracies was more likely in those who were SARS-CoV-2 vaccine hesitant and in ethnic minority groups [49]. Our findings confirm that misinformation relating to COVID-19 is important even amongst HCWs, and strategies to tackle this may increase vaccination uptake amongst HCWs and the population at large.

We found that those with evidence of previous COVID-19 were more likely to be vaccine hesitant than those who tested negative by PCR/serology [12]. This may reflect HCWs with evidence of previous SARS-CoV-2 infection feeling they have derived sufficient immunological protection against COVID-19 via natural infection and will therefore derive limited benefit from vaccination. Whilst this is likely to be true in a short period following the infective episode, over time, reinfection is possible. Population level data from Denmark indicate that infection with SARS-CoV-2 offers 80·5% protection against reinfection, dropping to 47·1% in those over 65 [50]. Furthermore, SARS-CoV-2 neutralising antibody dynamics in those recovered from COVID-19 have been shown to vary widely [51], and protective immunity to related seasonal coronaviruses is known to be short-lasting [52,53]. Therefore, HCWs with evidence of previous COVID-19 (particularly those who were infected many months previously) represent important targets for vaccination, and publicising this message in communications aimed at HCWs may improve uptake in this group.

Pregnancy was a strong predictor of vaccine hesitancy in our cohort. This may be because during the early phase of the vaccine rollout pregnant women were advised to delay receipt of SARS-CoV-2 vaccination until after delivery [54]. However, in light of the updated advice from JCVI that pregnant women should be offered vaccination against COVID-19, the increasing amount of safety data available for SARS-CoV-2 vaccination during pregnancy and the increased risk of severe COVID-19 associated with pregnancy, pregnant healthcare workers represent an identifiable group who should be targeted to ensure that the risks and benefits of vaccine uptake have been discussed [54,55].

We found some evidence that vaccine hesitancy was becoming less frequent as time moved forward which may indicate increasing confidence/experience in the UK vaccine programme although further work is required to understand this.

This is the largest study of SARS-CoV-2 vaccine attitudes in a multi-ethnic sample of UK HCWs at the start of a vaccine roll-out. The combination of quantitative and qualitative data provides an in-depth understanding of hesitancy among different ethnic groups. Despite these strengths, our study also has a number of limitations. There was the potential for self-selection/responder bias and we were not able to directly compute response rates by different ethnic groups due to information governance processes but the cohort respondents’ characteristics were broadly similar to the wider NHS workforce thus indicating that our sample is likely to be representative, albeit with a smaller proportion of ancillary staff. Overall the cohort included a relatively small number of ancillary staff. Similarly, we used the Office for National Statistics 18 ethnicity categories to provide more granular, policy-relevant, information and this resulted in some of the ethnicity categories having small numbers. Due to the rapidly evolving nature of the vaccination programme, questions relating to vaccination were changed midway through the baseline questionnaire rollout which could have impacted on outcome, although we have controlled for questionnaire version in the multivariable analysis. Our outcome measure of vaccine hesitancy is based upon the WHO's definition of refusal or delay in vaccine acceptance which means that some individuals who initially delayed acceptance but then went on to receive the vaccine would still be classified as hesitant. We think this is valid as HCWs delaying vaccine uptake during a global pandemic should be classed as hesitant. This may slightly overestimate the degree of hesitancy but we think the effect is likely to be minimal as our overall figure for hesitancy is in keeping with other published reports. The relevant sections of the baseline questionnaire were not designed to capture actual vaccine uptake as an outcome but rather attitudes towards vaccination and thus we cannot determine whether access to vaccination could be a driver in vaccine hesitancy in our sample, however this will be captured in follow-up questionnaires.

In summary, we have identified key predictors of SARS-CoV-2 vaccine hesitancy in HCWs and demonstrate clear ethnic differences in hesitancy levels. Importantly, we have established drivers behind vaccine hesitancy in HCWs, which include belief in COVID-19 conspiracies and mistrust (of vaccines in general, in SARS-CoV-2 vaccines specifically, in healthcare systems and research) and suggest that these factors may account for some of the observed ethnic differences in hesitancy. Strategies to improve vaccine confidence are urgently required to prevent these ethnic disparities from widening. Such strategies may include building trust and involvement/engagement of ethnic minority HCWs in the vaccination rollout, promoting vaccination and overcoming misinformation utilising trusted networks in ethnic minority communities.

## Contributors

MP conceived of the idea and led the application for funding with input from MDT, KK, ICM, KW, RF, LBN, SC, KRA, LJG, ALG and CJ. The survey was designed by KW, MP, ICM, CMel, CJ, ALG, LBN, RF and CAM. Online consent and survey tools were developed by LB. KW, CAM, ICM, and LBN wrote the first draft of the manuscript with input from MP and all co-authors. All authors approved the submitted manuscript.

## Data sharing

To access data or samples produced by the UK-REACH study, the working group representative must first submit a data and material request form to the Data Access Committee (DAC) providing details for all manuscript proposals. The DAC will establish priorities for core and ancillary projects. For ancillary studies outside of the core deliverables, the Steering Committee will make final decisions once they have been approved by the Core Management Group and the DAC. Decisions on granting the access to data/materials will be made within eight weeks. Third party requests from outside the Project will require explicit approval of the Steering Committee once approved by the Core Management Group and the DAC.

## Transparency statement

The lead authors affirm that this manuscript is an honest, accurate, and transparent account of the study being reported; that no important aspects of the study have been omitted; and that any discrepancies from the study as planned (and, if relevant, registered) have been explained.

## Funding

UK-REACH is supported by a grant from the MRC-UK Research and Innovation (MR/V027549/1) and the Department of Health and Social Care through the National Institute for Health Research (NIHR) rapid response panel to tackle COVID-19.

Core funding was also provided by NIHR Biomedical Research Centres.

KW is funded through an NIHR Career Development Fellowship (CDF-2017-10-008).

CAM is an NIHR Academic Clinical Fellow (ACF-2018-11-004).

LBN is supported by an Academy of Medical Sciences Springboard Award (SBF005\1047).

ALG was funded by internal fellowships at the University of Leicester from the Wellcome Trust Institutional Strategic Support Fund (204801/Z/16/Z) and the BHF Accelerator Award (AA/18/3/34220).

MDT holds a Wellcome Trust Investigator Award (WT 202849/Z/16/Z) and an NIHR Senior Investigator Award.

KK is supported by the National Institute for Health Research (NIHR) Applied Research Collaboration East Midlands (ARC EM).

KK and MP are supported by the NIHR Leicester Biomedical Research Centre (BRC).

MP is supported by a NIHR Development and Skills Enhancement Award.

This work is carried out with the support of BREATHE -The Health Data Research Hub for Respiratory Health [MC_PC_19004] in partnership with SAIL Databank. BREATHE is funded through the UK Research and Innovation Industrial Strategy Challenge Fund and delivered through Health Data Research UK.

## Disclaimers

The views expressed in the publication are those of the author(s) and not necessarily those of the National Health Service (NHS), the NIHR or the Department of Health and Social Care. This research was funded in whole, or in part, by the Wellcome Trust [WT204801/Z/16/Z and WT 202849/Z/16/Z]. For the purpose of open access, the author has applied a CC BY public copyright licence to any Author Accepted Manuscript version arising from this submission.

## Declaration of interests

KK is Director of the University of Leicester Centre for Black Minority Ethnic Health, Trustee of the South Asian Health Foundation, Chair of the Ethnicity Subgroup of the UK Government Scientific Advisory Group for Emergencies (SAGE) and Member of Independent SAGE. SC is Deputy Medical Director of the General Medical Council, UK Honorary Professor, University of Leicester. MP reports grants from Sanofi, grants and personal fees from Gilead Sciences and personal fees from QIAGEN, outside the submitted work. KW, ICM, CAM, LBN, ALG, CM, LB, MG, FW, AAO, OH, AG, CJ, MDT, SS, BG, AA, SZ, RR and LJG have no competing interests to declare.

## Appendix Supplementary materials

Image, application 1

## Footnotes

1 The ‘White Other’ group includes the ‘White Other’ and ‘White Gypsy and Irish Traveller’ categories, because there were very small numbers in the latter group.

Supplementary material associated with this article can be found in the online version at https://doi.org/10.1016/j.lanepe.2021.100180.
